# Supplementary material for: All-at-once RNA folding with 3D motif prediction framed by evolutionary information
Source: Nat Methods. 2025 Oct 3;22(10):2094–106. doi: 10.1038/s41592-025-02833-w (PMC12510878; doi:10.1038/s41592-025-02833-w)
Supplement: Supplementary file 3 — Source code to run CaCoFold-R3D. Data and results. [file 41592_2025_2833_MOESM3_ESM.gz › supplemental_material/results/CaCoFold-R3D/CaCoFold-R3D_Rfam_vshuffle/RF01573_DdR5.R2R.sto.pdf]

RF01573_DdR5

5′ - U C U A U G A U G A G U A U C G G A C A C U U U U G A A C U U A ● U U G A A A A C R A A A C A C G A U U U U U U G C G U U A U C U A A ● ● U U U Y C ● C U G A C A
